# Supplementary figures and images for: Novel regulators of PrPC biosynthesis revealed by genome-wide RNA interference
Source: PLoS Pathog. 2021 Oct 27;17(10):e1010013. doi: 10.1371/journal.ppat.1010013 (PMC8575309; doi:10.1371/journal.ppat.1010013)

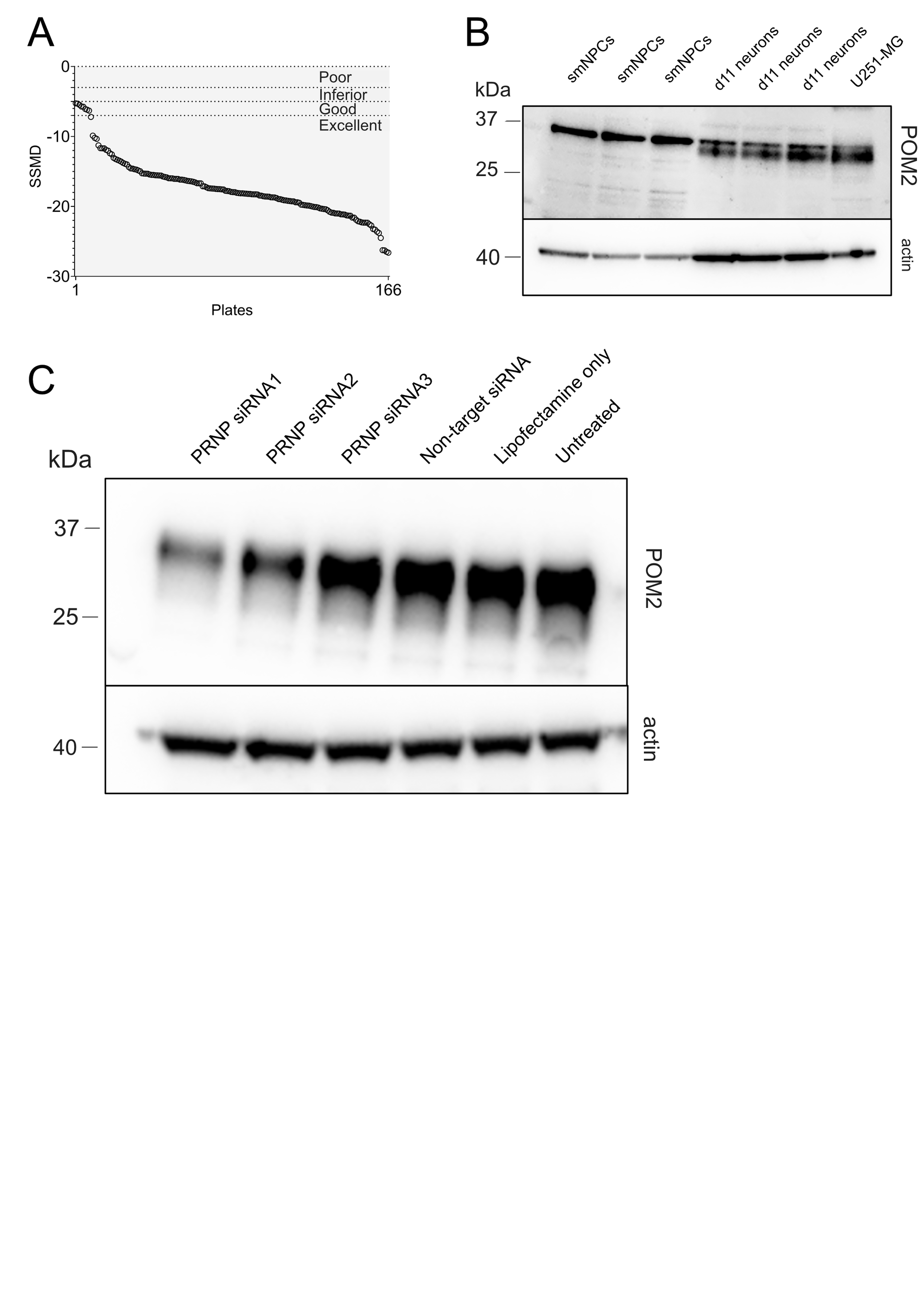

Supplement: S1 Fig — (A) Strictly standardized mean difference of each plate from the primary screening reporting the separation of the positive and negative controls. Range of the quality metrics (poor to excellent) is reported based on the highest level of stringency [25]. (B) Endogenous PrPC levels in human smNPCs and smNPC-derived neurons (day 11 post differentiation) assessed through immunoblotting. U251-MG was used as a positive control. The anti-PrP antibody POM2 was used for detection of PrPC. (C) Western blot analysis of 11 days old smNPC-derived neurons upon transfection with three distinct PRNP targeting siRNAs and a non-targeting siRNA as well as Lipofectamine only and untreated control. PRNP siRNA 1 and 2 lead to a marked decrease of PrPC levels 6 days after transfection. (TIF) [file ppat.1010013.s001.tif]

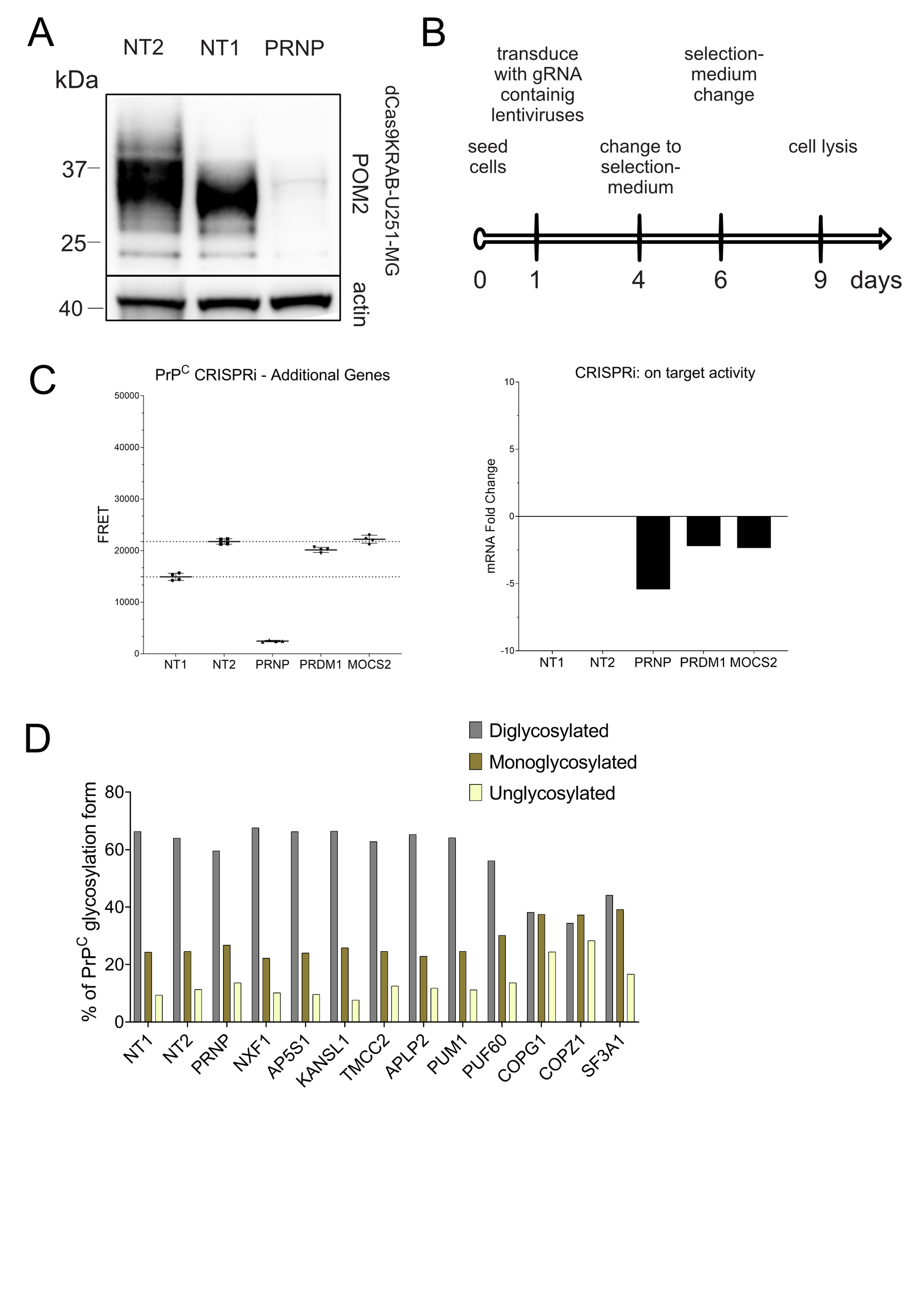

Supplement: S2 Fig — (A) Efficacy of CRISPRi in dCas9-KRAB-U251-MG through lentiviral transduction of a construct containing four single guides against PRNP as well as two non-targeting controls assessed through immunoblotting. After CRISPRi treatment followed by antibiotic selection for five days an evident reduction of PrPC levels is apparent. (B) Schematic depicting the setup of the CRISPRi experiments in dCas9-KRAB-U251-MG cells. (C) Two candidates tested independently. PrPC protein levels of dCas9-KRAB-U251-MG cells transduced with gRNA CRISPRi lentiviruses against both targets. Mean values ± SD (n = 4 technical replicates) are shown. CRISPRi activity measured by the mRNA level of the target gene in comparison to a negative control sample (NT1) after normalization to the housekeeping gene ACTB. (D) Quantification of the glycosylation pattern differences in all samples indicates a shift of glycosylation of PrPC upon regulation of PUF60, COPZ1, COPG1 and SF3A1. (TIF) [file ppat.1010013.s002.tif]

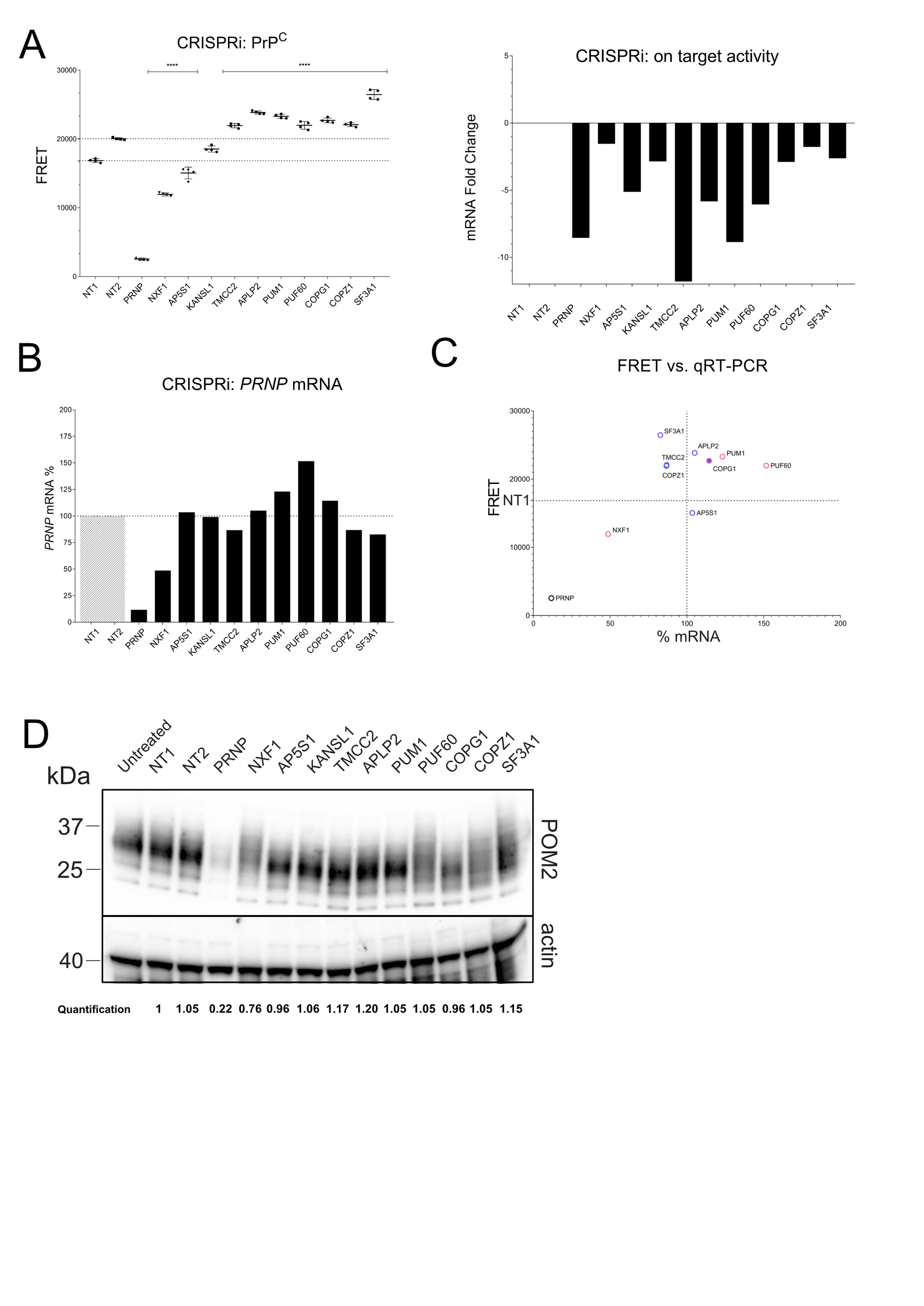

Supplement: S3 Fig — (A) Panels depicting the effect of CRISPRi downregulation on PrPC levels assayed using TR-FRET, as well as the on-target efficiency of CRISPRi downregulation on each target assessed by qRT-PCR. (B) Effect of CRISPRi downregulation in A assessing PRNP mRNA levels. ACTB was used as a housekeeping gene and samples were compared to non-target 1 (NT1) (C) Modulation of PRNP mRNA and PrPC protein levels after CRISPRi mediated suppression of 2 stabilizers and 7 limiters. qRT-PCR and PRNP mRNA FRET analysis of the nine hits with a significant effect on PrPC, in an independent repetition experiment. Samplers were normalized to ACTB. Three genes (pink) regulated PRNP mRNA levels, whereas five (blue) modulated PrPC post-transcriptionally and one gene (pink circle with blue filling) acted both transcriptionally and post-transcriptionally. (D) Western blot analysis of same samples as in A, probed with antibody POM2 against PrPC. FRET and immunoblot results were congruent and the shift in the glycosylation for PUF60, COPZ1, COPG1 and SF3A1 was persistent confirming the results from Fig 3C. For quantification, signal intensity of PrPC was normalized to the signal intensity of β-actin and compared to NT1. (TIF) [file ppat.1010013.s003.tif]

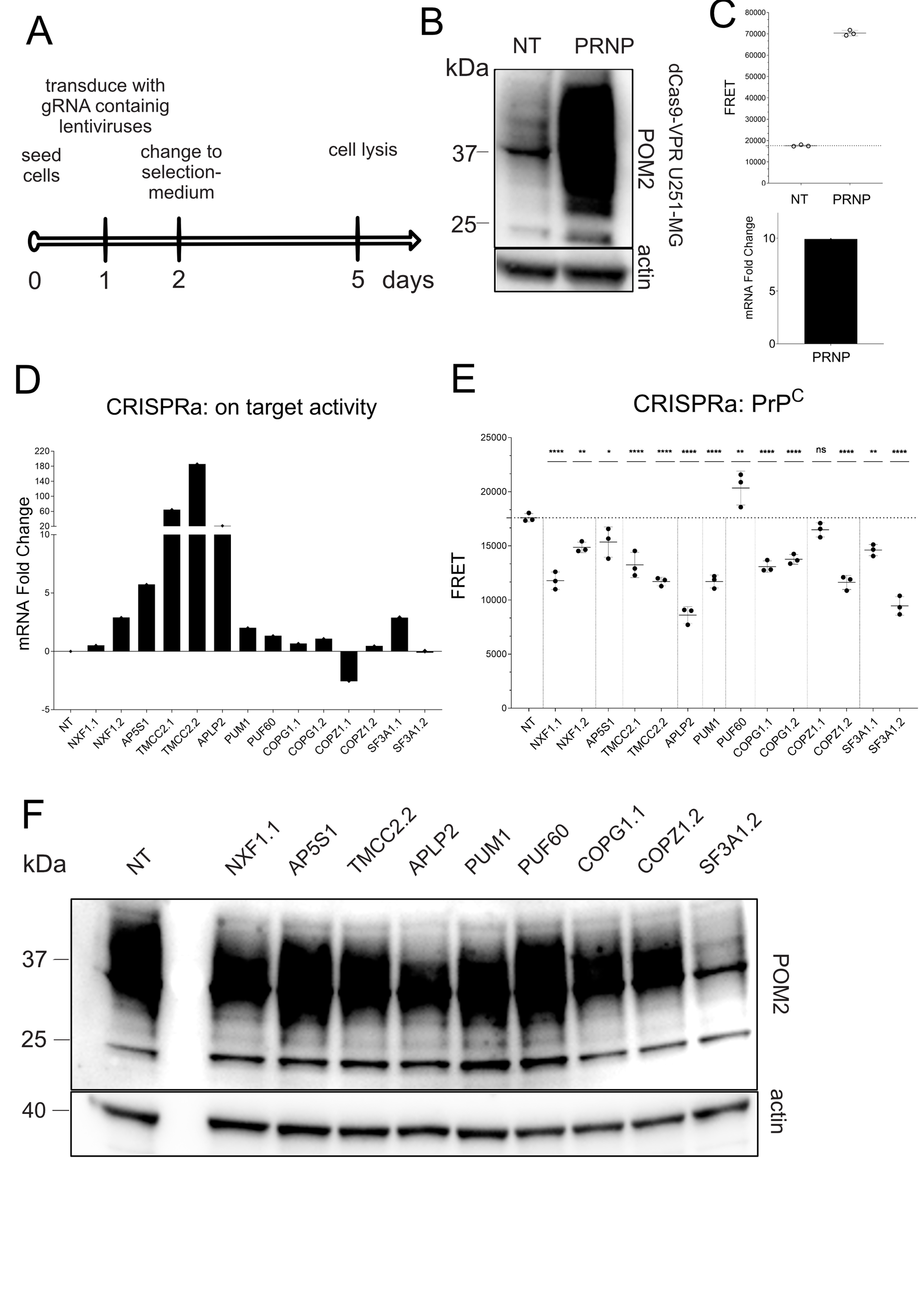

Supplement: S4 Fig — (A) Schematic depicting the setup of the CRISPRa experiments in dCas9-VPR-U251-MG cells. (B) Efficacy of CRISPRa in dCas9-VPR-U251-MG by lentivirally transduce a construct containing gRNAs against PRNP as well as a non-targeting control assessed through immunoblotting. (C) Additional quantification of B using TR-FRET. qRT-PCR of the same setup as in B. ΔCt values were normalized to those of ACTB. After CRISPRa treatment followed by antibiotic selection for three days an increase in PrPC levels is apparent. (D) CRISPRa efficiency for each gene measured by qRT-PCR after selection for three days. ΔCt values were normalized to those of ACTB. (E) Effect of CRISPRa for all targets assayed on PrPC levels measured by TR-FRET. (F) Western blot analysis of representative samples from E. Antibody POM2 was used for detection of PrPC. * p ≥ 0.05, ** p ≥ 0.01, **** p ≥ 0.0001, n.s. = non-significant (Dunnett’s multiple comparisons test). (TIF) [file ppat.1010013.s004.tif]
